# Colloid-Mediated Transport of Pharmaceutical and Personal Care Products through Porous Media

**DOI:** 10.1038/srep35407

**Published:** 2016-10-13

**Authors:** Yingna Xing, Xijuan Chen, Xin Chen, Jie Zhuang

**Affiliations:** 1 Key Laboratory of Pollution Ecology and Environmental Engineering, Institute of Applied Ecology, Chinese Academy of Sciences, Shenyang 110016, China; 2 University of Chinese Academy of Sciences, Beijing 100049, China; 3 Department of Biosystems Engineering and Soil Science, Institute for a Secure and Sustainable Environment, The University of Tennessee, Knoxville, TN 37996, USA

## Abstract

Pharmaceutical and personal care products (PPCPs) enter soils through reclaimed water irrigation and biosolid land applications. Colloids, such as clays, that are present in soil may interact with PPCPs and thus affect their fate and transport in the subsurface environment. This study addresses the influence of soil colloids on the sorption and transport behaviors of PPCPs through laboratory column experiments. Results show that the affinities of PPCPs for colloids vary with their molecular chemistry and solution ionic strength. The presence of colloids promotes the breakthrough of ciprofloxacin (over 90% sorbed on colloids) from ~4% to 30–40%, and the colloid-facilitated effect was larger at lower ionic strength (e.g., 2 mM). In comparison, the net effect of colloids on the transport of tetracycline (~50% sorbed on colloids) could be facilitation or inhibition, depending on solution chemistry. This dual effect of colloids is primarily due to the opposite response of migration of dissolved and colloid-bound tetracycline to the change in solution ionic strength. Colloids could also facilitate the transport of ibuprofen (~10% sorbed on colloids) by ~50% due likely to exclusion of dispersion pathways by colloid straining. This study suggests that colloids are significant carriers or transport promoters of some PPCPs in the subsurface environment and could affect their off-site environmental risks.

Pharmaceutical and personal care products (PPCPs) are widely present in the natural environment due to high rates of production and usage^1^. Their potential risks of toxicity to organisms cause increasing concern^2^. Pharmaceuticals cannot be completely assimilated and utilized in the body, and excess amounts are eliminated by way of excretion in an unmetabolized form or as active metabolites^3^. Personal care products are also often used externally, and are discharged into wastewater treatment plants (WWTPs) directly. Due to their incomplete removal in WWTPs, they are widely present in reclaimed water and sludge^4^,^5^. As a result, PPCPs enter soils when reclaimed water and biosolids are applied for agricultural production.

After entering soils, PPCPs have a greater chance to interact with soil colloids, which are ubiquitous in the soil environment. Colloids usually have a strong and large adsorption capacity due to their large specific surface area and the high density of reactive sites^6^. Under certain environmental conditions, they could act as a second phase (in addition to liquid phase) and carry the sorbed pollutants long distances in the subsurface environment^7^. The colloid-facilitated transport of some strongly sorbing contaminants has aroused concern. Karathanasis (1999) reported that colloids could increase the breakthrough amount of copper and zinc by 5–50 times^8^. Similar phenomenon was observed for lead, cadmium, and radionuclides^7^,^9^,^10^. Colloids also demonstrated a potential for promoting the transport of organic pollutants, such as prochloraz, glyphosate, and atrazine^11^,^12^,^13^, but relevant studies on emerging organic chemicals are few, and we lack a mechanistic understanding of their transport behaviors compared with heavy metals.

Ion exchange and surface complexation are the main mechanisms that govern the sorption of organics on colloids^14^,^15^. The sorption process can be affected by solution chemistry (e.g., pH and ionic strength) through alteration of adsorbent surface charges, adsorbate ionization, competition of sorption sites, and change in interfacial forces^16^,^17^. Existing literature shows significant sorption of PPCPs on colloids, implying that the fate and transport of PPCPs may be influenced by soil colloids. Unfortunately, the majority of studies on the interactions between PPCPs and colloids were focused on the sorption interaction using purified colloids with a single component, and few studies have addressed the effect of natural colloids on the transport of PPCPs under different chemical conditions.

Ciprofloxacin and tetracycline are antibiotics widely used by human beings and animals. Ciprofloxacin is frequently detected with concentrations of hundreds ng/L in waste water^18^,^19^ and was barely degraded in soil^20^. Approximately 50–80% of the dosage of tetracycline is excreted as unmetabolized parent compounds through feces and urine^21^, which are quite persistent in soil^22^. For instance, its concentrations in soil could reach 19.9 ng/g in Shijiazhuang, China^23^. Ibuprofen is a non-prescription drug that is produced annually at the kiloton level worldwide and is widely used by humans at a high daily dose^24^. Ibuprofen has been found frequently present in effluents and sludge, and its concentration in reclaimed water and soil reached 4,060 ng/L and 318.5 ng/L, respectively, in Texas, USA^25^,^26^,^27^. Although these PPCPs are frequently detected in soils^28^,^29^, little is known about their transport behaviors in soils, particularly the effect of soil colloids on their environmental risks.

This study aimed to elucidate the effects of colloids on the transport of three different PPCPs (ciprofloxacin, tetracycline, and ibuprofen) in porous media. Batch and breakthrough experiments were conducted under different ionic strength conditions to quantify the influence of soil colloids on the transport of PPCPs. The results could improve the understanding of the mechanisms that govern the fate and transport of PPCPs in soils as well as the potential of soil colloids in mediating the off-site risks of PPCPs.

## Materials and Methods

### Chemicals

PPCPs, including ciprofloxacin (purity ≥ 98%), tetracycline (purity ≥ 98%), and ibuprofen (purity ≥ 98%), were purchased from Tokyo Chemical Industry (Tokyo, Japan). Reagents and all the other chemicals (such as NaCl) used for high performance liquid chromatography (HPLC) analysis were pure class distinctions. The basic structure and physicochemical properties of the target PPCPs are provided in Table 1.

### Porous medium and colloids

Colloids (≤1 μm) used in the study were extracted by sedimentation from the topsoil (0–20 cm) of a no-till farmland located in an Experimental Station of Agroecology, Shenyang, China (41°31′N,123°24′E). Specifically, sampled bulk soil was air-dried and passed through a 100-mesh nylon sieve. Then, ~60 g of the sieved soil was added to 1 L of deionized water for a 30-minute ultra-sonication. The soil suspension was thoroughly mixed in 1-L graduated cylinder for extracting colloids (<1 μm) using the Stokes Law-based sedimentation method. Finally, the colloid slurry was freeze-dried and stored in brown glass vials at room temperature (22 ± 1 °C) for use.

The porous medium used in the study was quartz sand (0.5–1.0 mm in grain diameter) purchased from Sinopharm Chemical Reagent Co., Ltd (Shanghai, China). Prior to use, the quartz sand was treated with a 1 M HNO_3_ solution at 80 °C for 8 hours to remove the organic or inorganic impurities it may carry. Then, the sands were rinsed with deionized water until electrical conductivity reached the level of deionized water. Finally, the sand was dried at 105 °C. The physicochemical properties of the colloids and sand are provided in Table 2.

### Batch experiments

All sorption experiments were conducted in 30 mL glass centrifugal tubes in NaCl solution at two ionic strengths (2 mM and 10 mM, pH 6.2). The initial concentrations of PPCPs and colloids for kinetic studies were 2 mg/L and 100 mg/L, respectively. Two control experiments were conducted simultaneously to check whether there was sorption of target PPCPs to the wall of the tube. A 1:5 mass ratio of solid to liquid was used for the sand experiments. The tubes were shaken in the dark to prevent light-induced decomposition at 200 rpm and 25 °C. Equilibration time was 0.25, 0.5, 1, 2, 4, 8, 12, and 24 hours, after which the suspensions were centrifuged at 4,480 × g for 15 minutes, and the supernatants were used for analysis without further treatment. The sorption amounts of PPCPs were calculated from the difference between the initial and equilibrium concentrations. All experiments were performed in triplicates.

### Breakthrough experiments

Column experiments were performed under saturated flow conditions to investigate the transport of PPCPs. The column system used in the study consisted of a stainless steel column (10 cm length, 1 cm inner diameter), a peristaltic pump, and a fraction collector. The columns were packed with the air-dried sand, and tapped down after each 1-cm increment with a stainless steel rod. A thin layer (~0.5 mm) of glass wool was placed at the top and bottom of the column to prevent possible blockage of the tubing by fine sand grains during the flow experiments. After closing the column with the top plate, dissolvable high-pressure CO_2_ gas was passed into the column for ~20 minutes to replace the air in the sand medium.

The sand columns were then saturated and flushed by injecting ~20 pore volumes of a degassed background solution (NaCl, 2 mM or 10 mM, pH 6.2) from the bottom of the column to reach equilibrium of flow and chemical conditions. This was followed by an experimental solution of the same ionic strength, which contained the three PPCPs or pre-equilibrated PPCP-colloid mixture. The concentrations of injected PPCPs were 2 mg/L, which was almost the lowest concentration to ensure effective HPLC detection (detection limit 0.07 mg/L) for all effluent samples after sorption of PPCPs on the sand. The solution was immediately pumped into the sand column after preparation at a constant flow rate of ~0.2 mL/min, equivalent to a Darcy velocity of ~12.5 cm/h (Table 2). The effluent was collected into 4-mL glass tubes in fractions of ~2.2 mL. The solutions containing colloids at 100 mg/L were pre-equilibrated for 24 hours prior to injection. Moreover, the PPCP solutions contained 0.4 mM NaBr, which served as a conservative tracer (Br^−^) for calculating the dispersion coefficient of the transport model. After injection of ~7 pore volumes of the PPCP solution, the columns were flushed with ~6 pore volumes of NaCl of the same ionic strength as the PPCP solution. Detailed experimental parameters are provided in Table 3.

After effluent collection from the column, an aliquot of 0.8 mL from each sample fraction was transferred to a HPLC vial and stored at −18 °C until analysis; the rest was stored at −4 °C for measurement of bromide concentrations using ion chromatography (IC). For the experiments involving soil colloids, the collected liquid samples were first used to determine the concentrations of colloids prior to the centrifugation at 4,480 × g for 15 minutes to obtain clear supernatants for HPLC/IC analysis. The colloid concentrations were determined from a calibration curve based on the turbidity measurements at 680 nm using spectrometer (Spectrum 722E, Shanghai, China). The ciprofloxacin and tetracycline carried by colloids in each sample were measured after solvent extraction. Specifically, 3 mL of phosphoric acid-triethylamine buffer solution (pH 2.5) with acetonitrile (77:23, v/v) was added to each glass tube and vortexed for ~1 minute. Then, the tubes were shaken at 200 rpm at 25 °C in the dark for 2 hours before the samples were centrifuged for analyzing PPCPs in the supernatants. Validation data of colloid-sorbed ciprofloxacin and tetracycline were gained by extracting five colloid samples from their suspensions after 24 hours equilibration at five levels of ciprofloxacin or tetracycline concentrations (1–5 mg/L). For each concentration, duplicate samples were extracted, and the extraction recovery of ciprofloxacin and tetracycline were 105.2% ± 2.1% and 81.8% ± 2.2%, respectively. Ibuprofen was not extracted because our preliminary experiments showed that ibuprofen sorbed on the colloids was not detectable. All column experiments were performed in replicates to test reproduction.

### Analysis

Bromide analysis was conducted using a Dionex ICS-900 series IC system (Dionex. Sunnyvale, USA) with an AS22 separator column (Dionex, 4 × 250 mm). Colloids were measured using a spectrophotometer (Spectrum 722E) at a wavelength of 680 nm. PPCPs in all samples were analyzed using a Waters 2695 Series HPLC coupled with a Waters 996 photodiode array detector (PAD). Aliquots of 20 μL were injected, and the analytes were separated on a C-18 column (150 × 4.6 mm, 5-μm particle size; Thermo Scientific). For the analysis of ciprofloxacin and tetracycline, the mobile phase used was a phosphoric acid-triethylamine buffer (25 mM, pH 2.5) and acetonitrile (77:23, v/v), with ultraviolet detection at 278 nm and 350 nm, respectively. The analysis of ibuprofen was done by utilizing a mobile phase of a sodium phosphate buffer (10 mM, pH 3) and acetonitrile (45:55, v/v) with detection at 220 nm. The flow rate for both analyses was 1.0 mL/minute. Samples were quantified using external standards in the range of 0.1–2 mg/L. The limit of detection was ~70 μg/L, which was three times the standard deviation of the standard sample measurement (the signal to noise ratio was about 5:1). Therefore, the analysis results smaller than 0.1 mg/L were inaccurate and only used for reference. All of the data were acquired and processed using MassLynx 4.1 software.

### Modeling

The sorption data obtained through the batch experiments was fitted to the following pseudo-second-order kinetics equation^17^,^30^.


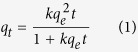


where k (g/mg/h) is the rate constant of sorption, q_e_ (mg/g) is the amount of PPCPs adsorbed at equilibrium, q_t_ (mg/g) is the amount of PPCPs adsorbed at time t, and kq_e_^2^ (g/mg/h) is the initial rate.

Hydrus-1D software was used to simulate the transport of total colloids and PPCPs in the packed columns. A one-dimensional form of the convection-dispersion equation with two kinetic retention sites was chosen^31^,^32^.


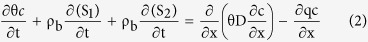


where t is time (h), θ is the volumetric water content (dimensionless), c is the concentration of colloid or PPCPs (mg/L), ρ_b_ is the bulk density of the sand (mg/cm^3^), x is the vertical spatial coordinate (cm), D is the hydrodynamic dispersion coefficient, q is the Darcy velocity (cm/h), and S_1_ and S_2_ are the solid phase concentrations associated with retention on Type 1 and 2 sites, respectively.

The two-site model assumes there are two fractions of the total sorption sites. Adsorption on the Type 1 site (Eq. 3) is assumed to be instantaneous and reversible, whereas the adsorption on the Type 2 site (Eq. 4) is kinetic and irreversible:


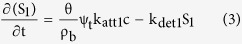



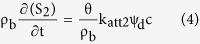


where k_att1_ (min^−1^) is the first-order attachment rate on Type 1 sites, k_det1_ (min^−1^) is the first-order detachment rate from Type 1 sites, k_att2_ (min^−1^) is the first-order attachment rate on Type 2 sites, and ψ_t_ and ψ_d_ are dimensionless functions to account for time-dependent retention and depth-dependent retention, respectively. The values of them are given as^33^,^34^:


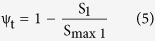



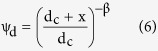


where S_max1_ is the parameter in the blocking function for Type 1 sites, β is an empirical factor controlling the shape of the spatial distribution, and d_c_ is the median diameter of the sand.

## Results and Discussion

### Sorption kinNetics

**Figure 1:**
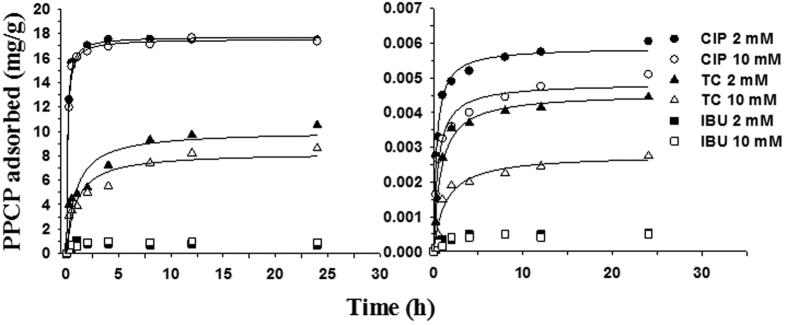
Sorption kinetics of PPCPs on colloids and sand. Symbols and lines represent measurement and simulation using the pseudo-second-order equation. CIP, ciprofloxacin; TC, tetracycline; IBU, ibuprofen.

Batch-scale kinetic studies were conducted to quantify the sorption and affinities of PPCPs on sand and soil colloids. Negligible sorption of PPCPs to the wall of the batch tubes was observed. Ciprofloxacin adsorbed on the colloids fastest and strongest among the three PPCPs. Its sorption reached equilibrium in ~4 hours (Fig. 1), faster than the reported results in the literature (8 hours)^35^. This discrepancy is assumed due to the high mass ratio (50:1) of colloids to ciprofloxacin as well as the surface chemistry disparity of colloids used in different studies. The *K*_d_ values of ciprofloxacin at the colloids were much larger (~8 × 10^4^ L/kg) than tetracycline (~1 × 10^4^ L/kg) and ibuprofen (~4 × 10^2^ L/kg) (Table 4). The equilibrium sorption of ciprofloxacin was similar at both ionic strengths (17,600 mg/kg at 2 mM and 17,500 mg/kg at 10 mM). The sand had ~4 orders of magnitude lower *K*_d_ values and equilibrium sorption for ciprofloxacin than the colloids due to their large differences in specific surface area and the density of high affinity sites. Increasing solution ionic strength decreased the sorption of ciprofloxacin to the colloids and the sand. This result is expected according to traditional electrochemical theory (i.e., increasing ionic strength is unfavorable to molecular sorption on oppositely charged surfaces)^17^. Because the pKa values of carboxylic group and amine group are 6.1 and 8.7^30^, respectively, part of the ciprofloxacin was protonated on the amine group in the piperazine moiety at pH 6.2 in this study. As a result, the dominant speciation of ciprofloxacin was zwitterion with positive charges^15^,^30^, while the sand was negatively charged. Worth noting is the much smaller effect of ionic strength on the sorption of ciprofloxacin at the colloids than at the sand. This difference is presumably attributed to the more complicated chemical interactions on the surface of colloids, which contain more organic matter than sand. At the sand, the sorption of PPCPs is mainly due to the cationic exchange mechanism^15^, which is subject to the variation of electrostatic forces with solution ionic strength. For colloids, non-electrostatic mechanisms (e.g., chemical complexation), which is relatively insensitive to ionic strength, might play a role in binding PPCPs^30^,^36^ in addition to cationic exchange.

Tetracycline showed slower and weaker sorption than ciprofloxacin on both the colloid and the sand. The sorption reached equilibrium in ~8 hours (Fig. 1). Its *K*_d_ values were nearly one order of magnitude lower at the colloids and nearly two times lower at the sand than those of ciprofloxacin (Table 4). The equilibrium sorption of colloids and sand for tetracycline were about half of those for ciprofloxacin under the same conditions. Increasing ionic strength decreased the sorption of tetracycline by 37.8% on the sand and 18.1% on the colloids. Similar to ciprofloxacin, this result is explainable because the pKa values of the tricarbonyl amide group, phenolic diketone group, and dimethylamino group in tetracycline are 3.3, 7.7, and 9.7 37, respectively. When there is sorption interaction, its pKa_1_ and pKa_2_ could shift to 6.23 and 8.01, respectively, due to protonation of tetracycline on the tricarbonyl amide group^37^,^38^. Thus, the dominant speciation of tetracycline at pH 6.2 in this study was a cationic molecule^39^. Electrostatic interaction dominated the sorption, leading to lower sorption of positively charged tetracycline at 2 mM and 10 mM.

Interestingly, ibuprofen did not show significant interactions with the colloids and the sand. This phenomenon is presumably due to the electric negativity of the dominant speciation of ibuprofen at pH 6.2, as reflected by its pKa of 4.9 40. The negative charges likely caused repulsion between ibuprofen and the negatively charged colloids and sand. However, this assumption is not confirmed by the negligible effect of ionic strength as observed in the experiments. It seems that neither electrostatic interactions nor surface chemical complexation plays a significant role in binding ibuprofen. Detailed mechanisms need further investigation.

To sum up, the affinities of the three PPCPs with the soil colloids and the sand are in order: ciprofloxacin > tetracycline > ibuprofen, and 24 hours is sufficient for reaching the equilibrium of sorption. The effect of ionic strength on the sorption depends on the dominant speciation of PPCPs in the solution.

### Effect of ionic strength on colloid transport

**Figure 2:**
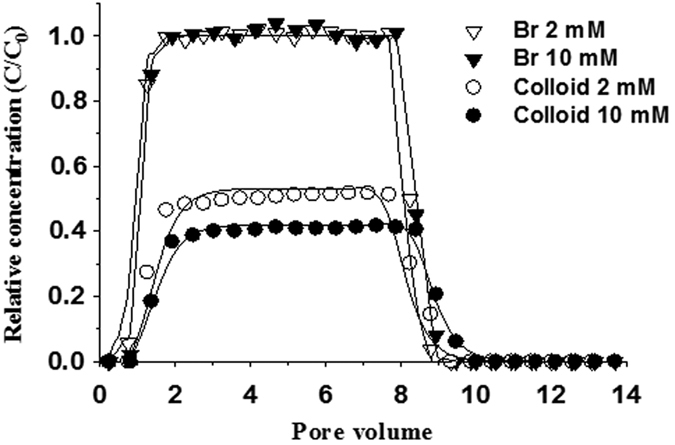
Breakthrough of bromide and colloids from sand under saturated flow conditions. Symbols and lines represent measurement and model fitting, respectively.

Solution ionic strength can influence the electric double-layer thickness of both colloid and sand and in turn change colloid mobility. Figure 2 shows the breakthrough curves of bromide as a conservative tracer and of soil colloids at different ionic strengths. The same curves of bromide from different experiments indicate the consistency of the experimental flow conditions. Increasing ionic strength from 2 mM to 10 mM decreased colloid transport through the sand column, with the maximum value of relative effluent concentrations (C/C_0_) decreasing from 0.52 to 0.41 and the recovery rates decreasing from 44.2% to 35.8% (Table 5). In pH neutral solution, both colloids and sand were negatively charged. The interactions of colloid-colloid and colloid-sand were dominated by electrostatic repulsive forces^41^, leading to considerable mobility of colloids. However, when the ionic strength increased, the electric double-layer was depressed to reduce the repulsive forces while increasing the overall attraction exerted by van der Waals forces^42^. Therefore, the deposition of colloids on other colloids or sand grains was enhanced, reducing the breakthrough of colloids from the sand column.

### Effect of ionic strength on transport of PPCPs

**Figure 3:**
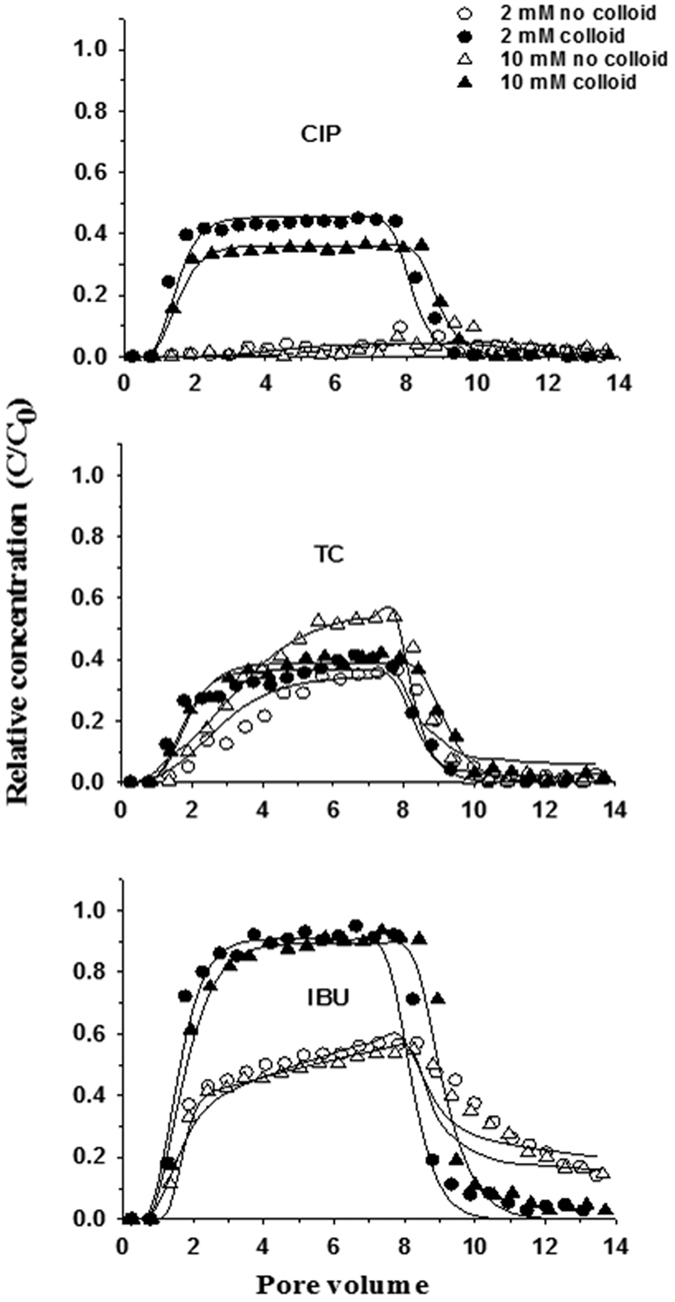
Breakthrough of total PPCPs from sand columns. Symbols and lines represent measurement and model fitting, respectively. CIP, ciprofloxacin; TC, tetracycline; IBU, ibuprofen.

Column experiments were conducted to investigate the effect of ionic strength on the transport of the three PPCPs. The breakthrough curves in Fig. 3 demonstrate that ciprofloxacin hardly broke through due to its high sorption affinity on the sand. Electrostatic attraction is the main interaction mechanism that increases the retention of ciprofloxacin containing cationic amine groups^43^. Increasing ionic strength had little influence on its transport as indicated by the minimal change in the values of k_att1_ and k_att2_ (Table 5). This insignificant effect is attributed to the overcoming of electrostatic interactions by non-electrostatic complexion interactions^43^.

Tetracycline had much higher mobility than ciprofloxacin (Fig. 3). The values of maximum C/C_0_ (0.37 at 2 mM and 0.54 at 10 mM) and recovery rates (24.1% at 2 mM and 35.4% at 10 mM) of tetracycline were significantly higher than those of ciprofloxacin. Solution ionic strength exerted significant effect on the transport of tetracycline, with the values of k_att1_ and k_att2_ decreasing significantly with increasing ionic strength (Table 5). These breakthrough results for tetracycline are consistent with its batch results.

Ibuprofen showed a high mobility, similar to tetracycline. However, increasing ionic strength did not significantly promote its retention on the similarly charged sand. Its maximum C/C_0_ values at both ionic strengths were similar (~0.55) (Table 5), and consistent with the batch results. This insignificant effect of ionic strength is attributed to the limited affinity of ibuprofen at the sand.

The above results suggest that tetracycline and ibuprofen have a higher off-site transport risk than ciprofloxacin, which could be mostly retained in soil^44^. The effect of ionic strength on the transport of PPCPs depends on the chemical speciation of PPCPs. Tetracycline moves more easily in solution with elevated concentrations and at neutral pH^17^,^32^. Our results imply that ionic strength effect could vary with solution pH. For instance, increasing ionic strength might reduce tetracycline mobility under alkaline conditions but increase it under acidic and neutral conditions.

### Coupled effect of ionic strength and colloids on the transport of PPCPs

Figure 3 shows the breakthrough of PPCPs at two ionic strengths in the presence and absence of colloids. The presence of colloids increased the recovery rate of ciprofloxacin by approximately one order of magnitude, i.e., from 3.9% to 39.5% at 2 mM and from 3.9% to 30.6% at 10 mM (Table 5). The values of both k_att1_ and k_att2_ of ciprofloxacin transport decreased significantly due to the colloid-facilitated effect. In the input solution, over 90% of ciprofloxacin was bound with colloids due to the high sorption affinity. As a result, colloids served as a mobile second phase (in addition to water) to carry ciprofloxacin through the sand^7^,^44^. The slightly higher recovery rate at 2 mM than 10 mM is attributed to the larger breakthrough of colloids at 2 mM^41^.

Colloids significantly enhanced the transport of ibuprofen by ~50%. Its recovery rates increased from 58.3% to 88.5% at 2 mM and from 57.0% to 85.1% at 10 mM when 100 mg/L colloids were present in the input solution. The significant decrease in the k_att2_ value suggests that the colloids reduced ibuprofen retention on the Type 2 sites (i.e., time-dependent irreversible sorption). The values of both k_att1_ and k_det1_ increased in the presence of colloids, with the magnitude of k_att1_ smaller than that of k_det1_. This result indicates that the instantaneous reversible retention on the Type 1 sites was approaching linear equilibrium conditions^45^. The colloid-facilitated transport of ibuprofen is attributed to colloid deposition or straining in the sand. The mechanically strained colloids blocked a portion of the pathways or covered some high-affinity sorption sites (e.g., the Type 2 sites) to reduce the dispersion and sorption of ibuprofen in the sand columns. Carrying by colloids did not contribute to the increased breakthrough of ibuprofen. This is because less than 10% of the ibuprofen in the input solution was adsorbed on the colloids after the 24-hour pre-equilibration, and the colloid-bound ibuprofen in the effluent samples was not detectable after centrifugation and solvent extraction. This small amount of binding of ibuprofen to the colloids suggests that the increased recovery rate in the presence of the colloids might be caused by the reduced ibuprofen-sand interactions^46^. Considering the minimal sorption of ibuprofen on the sand, we speculate that the deposited colloids blocked a portion of the pathways through which ibuprofen otherwise would disperse^41^,^47^.

Surprisingly, colloids did not show significant impact on the transport of tetracycline (Table 5 and Fig. 3). The values of k_att1_ and k_att2_ did not change significantly. This result is attributed to the weak sorption of tetracycline on the colloids. The minimal effect of ionic strength in the presence of colloids could result from the opposite tendencies of response of the transport of dissolved and colloid-bound tetracycline as ionic strength increased. At 2 mM, colloids reduced the retardation of tetracycline transport due to the sorption of tetracycline on the mobile colloids (Fig. 3). When the ionic strength increased to 10 mM, colloid mobility decreased (Fig. 2), weakening the potential for colloid-facilitated effect (Fig. 3)^42^. However, the amount of tetracycline in the liquid phase increased (i.e., lower sorption as shown in Fig. 1) due to its decreasing affinity with sand, and as a result tetracycline transport was enhanced (Fig. 3)^17^,^47^. The overall effect of colloids on the transport of tetracycline thus depends on both solution chemistry and colloid mobility. This result implies that tetracycline transport through clayey soils might vary greatly with environmental conditions.

### Environmental implications

Colloids can adsorb PPCPs and influence their transport behaviors in reclaimed water irrigated soils. For PPCPs that have high sorption (e.g., ciprofloxacin with K_d_ ~10^4–5^ L/kg) on soil colloids, the transport is dominantly controlled by colloids, with a higher colloid-facilitated effect at lower solution ionic strengths (e.g., 2 mM). This result implies that colloids might play a major role in carrying PPCPs to groundwater during irrigation or rainfall events. For PPCPs that have intermediate sorption (e.g., tetracycline with K_d_ ~10^3–4^ L/kg) on soil colloids, the mobility of dissolved and colloid-bound PPCPs responds oppositely to the change in solution ionic strength, making the net effect of soil colloids on PPCP transport vary with soil aqueous chemistry. Considering that many PPCPs have intermediate affinity with soil colloids, this result implies that adjustment of the reclaimed water chemistry might reduce the environmental transport of the majority of PPCPs. For the PPCPs with low sorption (e.g., ibuprofen with K_d_ ~10^2–3^ L/kg) on soil colloids, the presence of colloids increases PPCP transport due to reduced PPCP-sand interactions (e.g., pathway exclusion) by colloid deposition. This result implies that other measures (such as pre-filtration and surface modification) must be taken to reduce the environmental risks of low affinity PPCPs. Overall, this study suggests that the off-site migration risks of PPCPs are subject to their specific interactions with colloids and soil media and that it is necessary to include colloid-mediation in transport models. The study provides a significant insight into how to simultaneously control the environmental pollution risks of various PPCPs that are mixed in the reclaimed water.

## Additional Information

**How to cite this article**: Xing, Y. *et al*. Colloid-Mediated Transport of Pharmaceutical and Personal Care Products through Porous Media. *Sci. Rep.* **6**, 35407; doi: 10.1038/srep35407 (2016).

## Figures and Tables

**Table 1:**
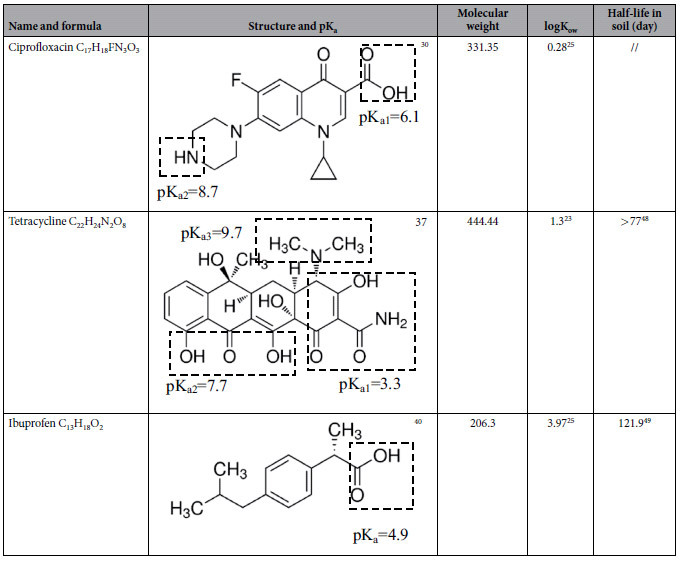
Structure and physicochemical properties of target PPCPs.

**Table 2:** Physicochemical properties of colloid and sand.

Materials	OM^a^	CEC^b^	Fe_d_^c^
	%	cmol/kg	g/kg
Colloid	1.14	48.1	16.5
Sand	−d	0.01	—

^a^Organic matter content determined by potassium dichromate oxidation method^50^.

^b^Cation exchange capacity at pH 7 determined by ammonium acetate method^51^.

^c^Free Fe oxide content measured by citrate–bicarbonate–dithionite method^52^.

^d^Not detected.

**Table 3:** Experimental conditions of experimental columns.

Ionic strength	Colloid	Bulk density	Soil porosity	Darcy velocity
mM	mg/L	g/cm	%	cm/h
2	0	1.54 ± 0.01	42	12.6 ± 0.04
2	100	1.54 ± 0.01	42	12.5 ± 0.05
10	0	1.53 ± 0.01	42	12.5 ± 0.09
10	100	1.54 ± 0.01	42	12.4 ± 0.04

**Table 4:** *K*_d_ values and sorption of PPCPs on sand and colloids after 24-hour equilibrium.

PPCP	2 mM				10 mM			
	*K*_d_ (L/kg)		Equilibrium sorption (mg/kg)		*K*_d_ (L/kg)		Equilibrium sorption (mg/kg)	
	Colloid	Sand	Colloid	Sand	Colloid	Sand	Colloid	Sand
CIP	79,047 ± 2,357	7.1 ± 0.33	17,600 ± 70	6.1 ± 0.11	71,759 ± 2,175	4.7 ± 0.23	17,500 ± 70	5.1 ± 0.11
TC	10,035 ± 155	4.2 ± 0.77	10,500 ± 70	4.5 ± 0.42	7,033 ± 104	1.9 ± 0.07	8,600	2.8 ± 0.07
IBU	390 ± 38	0.28 ± 0.02	750 ± 70	0.53 ± 0.04	443 ± 38	0.24 ± 0.04	850 ± 70	0.45 ± 0.07

Mean values  ±  standard errors are presented. CIP, ciprofloxacin; TC, tetracycline; IBU, ibuprofen.

**Table 5:** Transport parameters, maximum relative concentration (max C/C_0_), and recovery rate.

Parameter	2 mM without colloid			2 mM with colloid			
	CIP	TC	IBU	CIP	TC	IBU	Colloid
k_att1_ (min^−1^)	2.41E-01 ± 5.86E-02	5.80E-02 ± 1.33E-02	5.32E-02 ± 5.18E-03	2.15E-01 ± 6.77E-02	5.09E-02 ± 1.05E-02	2.20E-01 ± 6.03E-02	1.59E-01 ± 1.24E-02
k_det1_ (min^−1^)	2.69E-02 ± 2.44E-02	6.41E-04 ± 7.34E-04	6.24E-03 ± 6.79E-04	3.48E-01 ± 9.60E-02	3.22E-02 ± 1.19E-02	2.88E-01 ± 6.90E-02	1.92E-01 ± 1.44E-02
k_att2_ (min^−1^)	8.46E-01 ± 1.89E-03	1.79E-01 ± 5.92E-02	1.74E-01 ± 4.82E-02	2.13E-01 ± 1.89E-03	1.45E-01 ± 9.7E-03	2.41E-02 ± 2.11E-03	1.73E-01 ± 1.53E-03
R^2^	0.885	0.924	0.923	0.976	0.950	0.983	0.979
max C/C_0_	0.07 ± 0.01	0.37 ± 0.01	0.57 ± 0.02	0.45 ± 0.01	0.41 ± 0.01	0.94 ± 0.02	0.52 ± 0.02
recovery (%)	3.87 ± 0.01	24.1 ± 0.18	59.7 ± 0.93	39.5 ± 0.08	31.4 ± 0.57	88.5 ± 1.7	44.2 ± 1.13
Parameter	10 mM without colloid			10 mM with colloid			
	CIP	TC	IBU	CIP	TC	IBU	Colloid
k_att1_ (min^−1^)	2.85E-01 ± 2.86E-02	3.45E-02 ± 1.60E-03	4.87E-02 ± 1.57E-03	2.13E-01 ± 4.79E-02	1.23E-01 ± 3.27E-02	1.75E-01 ± 2.92E-02	2.08E-01 ± 2.24E-02
k_det1_ (min^−1^)	2.23E-02 ± 1.77E-02	1.55E-03 ± 7.46E-04	4.28E-03 ± 4.51E-04	2.86E-01 ± 5.32E-02	9.57E-02 ± 2.41E-02	1.79E-01 ± 2.62E-02	3.08E-01 ± 3.35E-02
k_att2_ (min^−1^)	7.76E-01 ± 1.64E-01	1.17E-01 ± 9.15E-03	2.17E-01 ± 2.05E-01	2.60E-01 ± 1.29E-03	1.39E-01 ± 2.33E-03	2.77E-02 ± 1.54E-03	2.23E-01 ± 7.98E-04
R^2^	0.873	0.948	0.931	0.992	0.985	0.986	0.995
Max C/C_0_	0.09 ± 0.01	0.54 ± 0.03	0.55 ± 0.03	0.38 ± 0.01	0.41 ± 0.03	0.93 ± 0.01	0.41 ± 0.03
Recovery (%)	3.92 ± 0.02	35.4 ± 0.45	57.0 ± 1.27	30.6 ± 0.11	33.5 ± 0.27	85.1 ± 1.37	35.8 ± 0.8

Mean values ± standard errors are presented. k _att1_, the first-order retention coefficient on Type 1 site; k_det1_, the first-order detachment coefficient on the Type 1 site; k_att2_, the first-order detachment coefficient on the Type 2 site; R^2^, Person’s squared correlation coefficient; CIP, ciprofloxacin; TC, tetracycline; IBU, ibuprofen.
